# Supplementary material for: Identification and functional analysis of non-coding regulatory small RNA FenSr3 in Bacillus amyloliquefaciens LPB-18
Source: PeerJ. 2023 May 15;11:e15236. doi: 10.7717/peerj.15236 (PMC10194069; doi:10.7717/peerj.15236)
Supplement: Supplemental Information 4 [file peerj-11-15236-s004.zip › KO/CK-vs-T1_map/map05216.html]

KEGG PATHWAY: Thyroid cancer - Reference pathway


|  |  |
| --- | --- |
| **Thyroid cancer - Reference pathway** |  |

[
Pathway menu
| Organism menu
| Pathway entry
| Show description
| User data mapping
]

Thyroid cancer is the most common endocrine malignancy and accounts for the majority of endocrine cancer- related deaths each year. More than 95% of thyroid carcinomas are derived from follicular cells. Their behavior varies from the indolent growing, well-differentiated papillary and follicular carcinomas (PTC and FTC, respectively) to the extremely aggressive undifferentiated carcinoma (UC). Somatic rearrangements of RET and TRK are almost exclusively found in PTC and may be found in early stages. The most distinctive molecular features of FTC are the prominence of aneuploidy and the high prevalence of RAS mutations and PAX8-PPAR{gamma} rearrangements. p53 seems to play a crucial role in the dedifferentiation process of thyroid carcinoma.

|  |  |  |
| --- | --- | --- |
| Reference pathway | 184% 150% 122% 100% 82% 67% 55% | 图片下载 |
